# Computational modeling of the bHLH domain of the transcription factor TWIST1 and R118C, S144R and K145E mutants

**DOI:** 10.1186/1471-2105-13-184

**Published:** 2012-07-28

**Authors:** Amanda M Maia, João HM da Silva, André L Mencalha, Ernesto R Caffarena, Eliana Abdelhay

**Affiliations:** 1 Laboratório de Célula-tronco – CEMO/INCA, Praça da Cruz Vermelha 23 6 andar, Centro, Rio de Janeiro/RJ, Brasil; 2 Laboratório de Biofísica Computacional e Modelagem Molecular – PROCC/ FIOCRUZ, Av Brasil, 4365, Manguinhos, Rio de Janeiro/RJ, Brasil

**Keywords:** Twist1, Transcription factor, bHLH, Comparative modeling, Molecular dynamics simulation, Collective motions

## Abstract

**Background:**

Human TWIST1 is a highly conserved member of the regulatory basic helix-loop-helix (bHLH) transcription factors. TWIST1 forms homo- or heterodimers with E-box proteins, such as E2A (isoforms E12 and E47), MYOD and HAND2. Haploinsufficiency germ-line mutations of the **twist1** gene in humans are the main cause of Saethre-Chotzen syndrome (SCS), which is characterized by limb abnormalities and premature fusion of cranial sutures. Because of the importance of TWIST1 in the regulation of embryonic development and its relationship with SCS, along with the lack of an experimentally solved 3D structure, we performed comparative modeling for the TWIST1 bHLH region arranged into wild-type homodimers and heterodimers with E47. In addition, three mutations that promote DNA binding failure (R118C, S144R and K145E) were studied on the TWIST1 monomer. We also explored the behavior of the mutant forms in aqueous solution using molecular dynamics (MD) simulations, focusing on the structural changes of the wild-type versus mutant dimers.

**Results:**

The solvent-accessible surface area of the homodimers was smaller on wild-type dimers, which indicates that the cleft between the monomers remained more open on the mutant homodimers. RMSD and RMSF analyses indicated that mutated dimers presented values that were higher than those for the wild-type dimers. For a more careful investigation, the monomer was subdivided into four regions: basic, helix I, loop and helix II. The basic domain presented a higher flexibility in all of the parameters that were analyzed, and the mutant dimer basic domains presented values that were higher than the wild-type dimers. The essential dynamic analysis also indicated a higher collective motion for the basic domain.

**Conclusions:**

Our results suggest the mutations studied turned the dimers into more unstable structures with a wider cleft, which may be a reason for the loss of DNA binding capacity observed for *in vitro* circumstances.

## Background

*TWIST1* is essential in embryological morphogenesis, mesoderm patterning and development. The protein is highly conserved from *Drosophila* to humans. In vertebrates, *TWIST1* is involved in cell type determination and differentiation during myogenesis, cardiogenesis, neurogenesis [1], hematopoiesis [2] and osteogenesis [3]. TWIST1 is a basic helix-loop-helix (bHLH) transcription factor (TF) in which the basic DNA-binding region is followed by a dimerization region composed of two amphipathic α-helices separated by a loop domain. TWIST1 forms either homo- or heterodimers with other bHLH proteins and binds to short conserved sequences called E-boxes (5´-CANNTG–3´) in promoter regions, regulating the transcription of target genes [4].

The dimer partner choice is a critical factor in determining TWIST1 activity in both flies and vertebrates [5,6]. In mammals, the transcription of thrombospondin is induced by heterodimers of TWIST1 with E2A (also known as TCF3; it presents two isoforms, E12 and E47), whereas homodimers of TWIST1 up-regulate the transcription of FGFR2 and periostin. *In vitro* assays have shown that TWIST1/E2A heterodimers bind DNA more efficiently than their homodimers [7], and this association also protects TWIST1 from ubiquitin-dependent proteasome degradation [8]. The TWIST1/E2A heterodimer also represses osteoblast differentiation by downregulating the expression of CDKN1A (p21), an inhibitor of cyclin-dependent kinases [9]. It has been shown that heterodimers of MyoD with E12 or E47 bind to the E-box sequence more efficiently than E12 or even E47 homodimers [10]. As only the heterodimers of the myogenic bHLH protein with the ubiquitous E2A protein are able to activate muscle-specific gene expression and differentiation, it is very important to ensure that only these heterodimers, and not E2A protein homodimers, bind to the relevant E-box sites. The myogenic bHLH proteins do not form homodimers efficiently. To compete with the E2A protein homodimers, the heterodimers must have a higher affinity for the binding site. However, this does not mean that E2A protein homodimers are of no use. The E2A proteins in B cells may be unique in their ability to bind DNA as homodimers. In muscle cells and pancreatic cells, they clearly prefer to bind DNA as heterodimers [10-13].

Null mutations of *twist1* in *Drosophila* result in embryonic lethality because of the complete absence of mesoderm, and homozygous knock-out mice die at E10.5-11, presenting a failure of neural tube closure and defects in the head mesenchyme, branchial arches, somites and limb buds [14]. Mice that are heterozygous for *twist1* null mutations display a phenotype that is similar to a human hereditary disorder called Saethre-Chotzen Syndrome (SCS – also known as acrocephalosyndactyly type III). Humans with *twist1* gene germ-line haploinsufficiency suffer from premature fusion of cranial sutures, skull deformations, limb abnormalities and facial dysmorphism [15].

More than 70 different mutations in the *TWIST1* gene have been identified in unrelated SCS patients and cluster in the bHLH coding sequence, either truncating or disrupting the transcription factor [16,17]. Approximately 75% of these mutations are single base pair substitutions that either create premature termination codons or substitute highly conserved residues in the bHLH region. The first type of mutation is represented mainly by nonsense mutations that are upstream to or within the bHLH motif. These mutations produce truncated proteins that rapidly degrade. The second type of mutations are missense mutations that involve the helix I or II region, creating proteins that fail to heterodimerize and which then become abnormally located in the cytoplasm [18]. Three missense mutations described by El Ghouzzi [18], Arg118Cys (R118C – helix I), Ser144Arg (S144R - loop) and Lys145Glu (K154E – helix II), are important because they lead to a loss of DNA binding for the TWIST/E12 heterodimer and, as a result, impair TWIST1 activity.

The three-dimensional (3D) structure of the TWIST1 protein has not yet been solved experimentally, and as the structure and function of a protein are intimately correlated, the elucidation of the 3D structure of TWIST1 could allow function prediction studies and the possibility of studying mutation effects, dynamic behavior under different conditions, and rational drug design. Given that only a limited number of proteins have had their 3D structures solved, theoretical methods, such as *ab initio* or comparative modeling, would appear to be fast and reliable methods for addressing this issue.

Because of the importance of *TWIST1* in the regulation of embryonic development, its substantial relationship with SCS and the lack of an experimentally solved structure for this protein, we performed comparative modeling for the TWIST1 bHLH region for both the homodimer and heterodimer with E47. These are important for DNA binding in the promoter region of target genes, and we evaluated their behavior in aqueous solution using molecular dynamics simulations. Three mutations that promote DNA binding failure, R118C, S144R and K145E, were also studied.

## Methods

### TWIST1-bHLH dimer structure construction

The human TWIST1 sequence was obtained from the International Protein Index (IPI) database and was analyzed to identify the conserved domains and secondary structure using the *Eukaryotic Linear Motif* (ELM) resource for functional sites in proteins [19] and GlobPlot2 [20], respectively. The Globplot2 parameters that were used to suggest a disordered region were examined using the Russel/Linding propensity algorithm, which is based on the hypothesis that the tendency of amino acids to be disordered can be expressed by the difference between the propensity to be a “random coil” versus a regular “secondary structure”, as defined by DSSP. A search for TWIST1 homology sequences to identify a template for comparative modeling was performed using the BLASTp program (Basic Local Alignment Search Tool for proteins - [21]) with the BLOSUM62 comparison matrix and the RSCB Protein Databank [22]. Template selection was based on a high percentage of coverage combined with the best levels for identity and similarity. Sequence alignment between TWIST1 and the selected template was performed using the ClustalW2 program [23,24] and the default parameters for the local alignment.

The three-dimensional (3D) models for the TWIST1 homodimer (TWI/TWI), TWIST1/E47 heterodimer (TWI/E47) and monomeric TWIST1 mutated models R118C, S144R and K145E were built using the MODELLER 9v6 package [25]. One hundred models were randomly generated from the template structure for each model (wild-type and mutants). The model with the lowest Objective Function score, which is the sum of all of the restraints, was subjected, by MODELLER scripts, to a root mean square deviation (RMSD) analysis taking the constraints of the template as a reference. Optimization was performed using the variable target function method [26] and employing the conjugate gradient algorithm, along with molecular dynamics with simulated annealing, to relax the models. Comparative modeling using the MODELLER program was performed with a desktop computer with an Intel® Dual Core™ CPU (1.8 GHz) in a Windows operating system environment.

### Model analysis

The models were subjected to detailed evaluation and were checked for possible errors using the tools that were available for structural assessment at the SwissModel Workspace [27]. QMEAN6 [28] estimates the global model quality and returns a pseudo-energy value, which can be used to compare and rank alternative models of the same target, with the best model represented by the lowest predicted energy. PROCHECK assesses the stereochemical quality of a protein by analyzing Ramachandran plots [29]. DFIRE is an all-atom statistical potential analysis that aids in the evaluation of non-bonding atomic interactions. It generates pseudo-energy values for the entire model that reflect its quality. DFIRE can be used for ranking alternative predictions of the same target. The lowest pseudo-energy values indicate models that are closer to the native conformation [30]. The QMEAN Z-score corresponds to a measurement of the absolute quality of a model, providing an estimation of the “degree of nativeness” of structural features that are observed in a model, and describes the chance that a given model is of a quality that is comparable to experimental structures. Models with low quality are expected to have strongly negative QMEAN Z-scores [31].

### Molecular dynamics simulation and analysis

Molecular dynamics (MD) simulations were performed using the GROMACS package v. 4.5.3 [32] according to the following procedures. First, the best homology model for the dimers was inserted into a 60 Å x 60 Å x 80 Å TIP4P [33] solvated orthorhombic box, and the system was neutralized by adding negatively charged Cl^-^ counter ions at random positions. All of the simulations were performed using the OPLS-AA (Optimized Potentials for Liquid Simulations, including every atom explicitly) force field [34]. Periodic boundary conditions were applied in all directions. The final configuration of all of the systems is described in Additional file 1: Table S1. To remove highly repulsive contacts, the system was submitted to 1000 steps of energy minimization using the steepest descent method with GROMACS program. All of the bond lengths were constrained with the LINCS algorithm. Non-bonded interactions were taken into account using the 6–12 Lennard-Jones potential, using a cut-off radius of 14 Å and a PME electrostatic treatment with a 10 Å radius for the coulomb interactions. All of the MD simulations were performed in the Gibbs ensemble at 300 K and 1 atm using the Berendsen algorithm. The simulations were conducted in two steps: equilibration and trajectory collection. For the equilibration stage, 1 ns was performed with all atomic protein positions restrained. The second step was a simulation without restraints, performed for 50 ns. In both stages of the simulation, a 2-fs time step was applied [32]. All calculations were carried out on an Intel(R) Core(TM) i7 CPU (2.67 GHz) machine, and the GROMACS program ran under an MPI protocol with the jobs distributed to 8 processors.

Trajectories for both homo- and heterodimers in aqueous solution were analyzed to obtain structural and dynamic properties using the GROMACS analysis tools package, including the interaction potential energy between the monomers of the dimers, root mean square fluctuation of the residues (RMSF), root mean square deviation (RMSD) of the monomer backbone and for each region, radius of gyration (Rg) of the monomer backbone and each region, secondary structure prediction using the DSSP program for the dimers, minimum distance between the centers of mass between regions, variation of the solvent accessible surface area (SASA - ΔSASA was calculated by subtracting the sum of the SASAs of the individual monomers from the SASA of their respective dimers) and collective motions (essential dynamics using *g_covar* and *g_anaeig* analysis in the GROMACS package) during the simulation time. The porcupine plots were generated by the Dynatraj webserver [35] and plotted using VMD software [36].

## Results

### Construction of TWIST1 models

**Figure 1:**
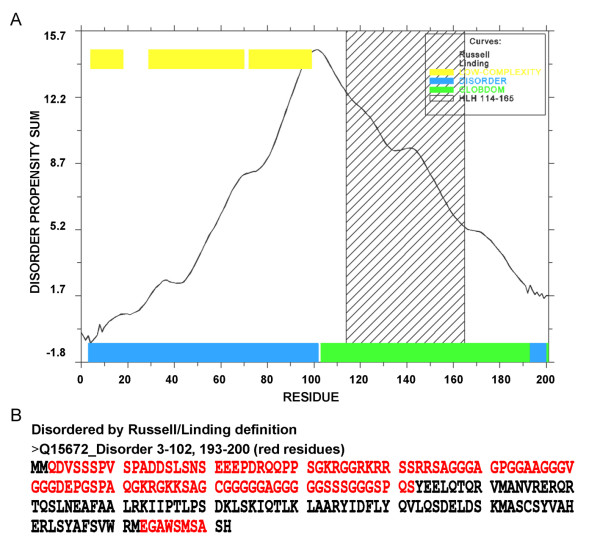
**Analysis of protein globularity. **(**A**) The x axis represents the sequence residues and the sum of disorder propensities are on the y axis. The yellow bars (upper left) correspond to residues with low structural complexity, the green bars correspond to the globular domain, and the blue bars represent the disordered residues. (**B**) The protein sequence is colored according to the Russel/Linding disorder definition, where the red residues have disorder propensity.

According to ELM, the human TWIST1 sequence deposited in IPI (IPI00018907) displayed three regions: the N-terminal region (residues 1–108), the bHLH domain (residues 109–163) and the C-terminal region (containing a Twist-box and WR motif comprising residues 164–202). The GlobPlot2 program determined that the N-terminal region is highly disordered (Figure 1), whereas the C-terminal region was identified as a disordered region that is intercalated with α-helical structures. As a consequence, the modeling of these domains was not performed in this study. The bHLH domain was the only segment of the TWIST1 sequence that presented a hit for proteins with known 3D structures according to BLASTp using the Brookhaven Database. The identity between the TWIST1_bHLH and E47/NeuroD1 sequences was 47%, with an *e-value* of 1e-08 (Additional file 2: Table S2). Therefore, the coordinates of the E47/NeuroD1 crystal (PDB code 2QL2 [37]) were chosen for modeling the bHLH domain.

**Figure 2:**
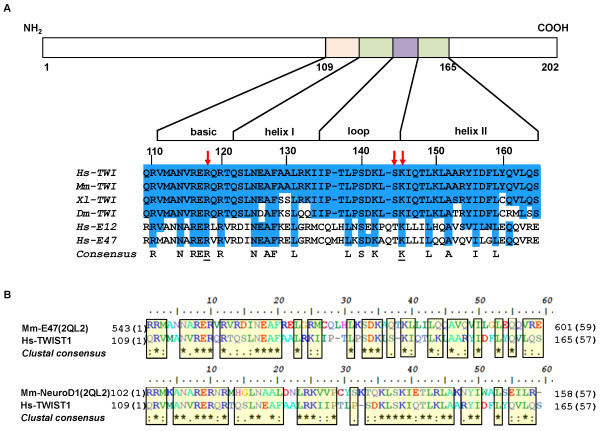
**Schematic representation of the human TWIST1 protein. **(**A**) Multiple sequence alignments for the bHLH domain of human TWIST1 and sequences belonging to different species. Conserved residues among species are highlighted in blue blocks. The modeled mutation positions R118C, S144R and K145E are indicated by red arrows. (**B**) Alignment between the TWIST1_bHLH sequence and the sequences that correspond to both chains of 2QL2 dimers (C – E47 and D – NeuroD1). All of the alignments were generated by ClustalW2 with default parameters and were plotted with the BioEdit program. The symbol ”*” indicates identical amino acids between sequences and “:” indicates conserved substitutions; “.” indicates semi-conserved substitutions. The yellow blocks highlight conserved residues. Hs – *Homo sapiens*; Mm – *Mus musculus*; Xl – *Xenopus laevis*; Dm – *Drosophila melanogaster*.

There is a high degree of conservation for human R118 and K145 in the bHLH protein family, which is illustrated by the alignment between different species of TWIST1_bHLH sequences and the sequence of human E2A protein (Figure 2A, *consensus*). Both amino acids appear underlined in the *consensus* sequence. Figure 2B illustrates the pairwise alignments between TWIST1_bHLH and the E47 and NeuroD1 monomers.

**Figure 3:**
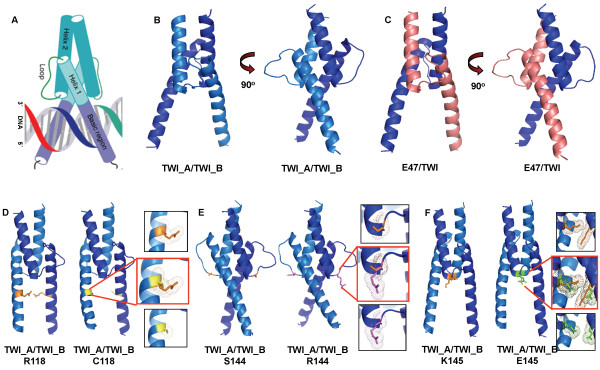
**Front and side view of the TWIST1 dimers. **(**A**) The cartoon representation of the plausible structure for the TWIST1 dimer in complex with DNA. (**B**) The cartoon representation of modeled dimers TWI_A/TWI_B (frontal and side views) and (**C**) the modeled dimer E47/TWI (E47 - pink frontal and side view). (**D**), (**E**) and (**F**) present the modeled homodimer TWI_A/TWI_B harboring the R118C, S144R and K145E mutations, respectively. The side chains of all three mutations are depicted in the side boxes.

The bHLH_TWIST1 dimer model, obtained by the arrangement of two monomers, was formed by two amphipathic α-helices that were separated by a loop region. The lowest “objective function” of the MODELLER program was used to select the best models for the wild-type and the R118C, S144R and K145E mutant forms. The scheme of the TWIST1 dimer in the complex with DNA and generated models of the homo- and heterodimers are shown in Figure 3 (A, B and C, respectively). At this point, the dimer models were not generated with DNA.

The structural changes promoted by the R118C, S144R and K145E mutations in the TWIST1 monomer were examined (Figure 3 D, E and F, respectively). The R118C substitution resulted in the neutralization of the net charge at this site. In addition, it is worth mentioning that both residues (arginine and cysteine) differed significantly in SAS, with a decrease of up to 90 Å^2^ of total surface accessible residue area (SAS – Additional file 3: Table S3). The substitution S144R also led to a modification in the AA charge (neutral to positive), with an enhancement of the solvent accessible surface area (SASA) of the mutated amino acid (SASA of 100 Å^2^ – Additional file 3: Table S3). Finally, the substitution K145E accounted for both homo- and heterodimer inversion of the charges (positive to negative). No evident difference was observed for SAS, and a shift to a more hydrophilic profile was observed (hydrophobic values changed from 125 to 40).

### Model validation

Stereochemical validation of all of the models was performed with the PROCHECK program and indicated that, after the MODELLER procedure minimization, they did not present aberrations. The Ramachandran plot of the template structure revealed that the K574 (chain C) amino acid was in a disallowed position. This error was propagated to the models that used the 3D structure of the E47 protein as a monomer, now corresponding to the K32 (chain A) residue in the models. All of the structures evidenced more than 99% of the residues in the allowed region of the Ramachandran plot. The modeled structures presented better values than the template structure, which presented 97% of the residues in the allowed regions (Table 1). This observation likely results from the minimization energy treatment of the modeled dimers.

**Table 1:** Summary from SWISSMODEL evaluation package pseudo-energies

		***E47/NeuroD1***	**TWI_A/TWI_B wt**	**TWI_A/TWI_B R118C**	**TWI_A/TWI_B S144R**	**TWI_A/TWI_B K145E**	**E47/TWI wt**	**E47/TWI R118C**	**E47/TWI S144R**	**E47/TWI K145E**
ENERGY ANALYSIS	DFIRE	***-138.26***	-143.02	-144.22	-144.23	-147.04	-146.4	-143.02	-146.87	-148.82
	QMEAN6	***0.668***	0.762	0.757	0.806	0.804	0.854	0.835	0.840	0.855
	Z-score	***-0.98***	0.02	**-0.03**	0.47	0.45	**-0.93**	0.73	0.79	0.94
RAMACHANDRAN PLOT	Most favored	***95.2%***	96.2%	97.1%	96.2%	97.1%	97.2%	95.3%	96.3%	96.3%
	Allowed	***1.9%***	2.9%	2.9%	2.9%	2.9%	1.9%	3.7%	2.8%	2.8%
	Generously allowed	***1.9%***	1.0%	0%	1.0%	0%	0%	0%	0.9%	0.9%
	Disallowed	***1.0% (K574/C)***	0%	0%	0%	0%	**0.9%** (K32/A)	**0.9%** (K32/A)	0%	0%

The bad scores are in bold, and the template scores are in bolditalic column. For the Ramachandran plot, the percentages were related to the amount of residues in each area of the plot. TWI – TWIST1 monomer; wt – wild-type.

According to the DFIRE and QMEAN6 analyses, which evaluated the model analyzing non-bonded atomic interactions and global model quality, respectively, all of the models presented score values that were higher than the template (−138.26 and 0.668, respectively), as shown in Table 1. The QMEAN6 Z-score values also confirmed that modeled proteins improved their three-dimensional structure, presenting values that were higher than the template (−0.98). The only structure that presented a similar score to the template was the structure that corresponded to the wild-type (wt) heterodimer (−0.93), which was likely influenced by the template structure (Table 1).

### Molecular dynamic simulations of wild-type and mutant proteins

**Figure 4:**
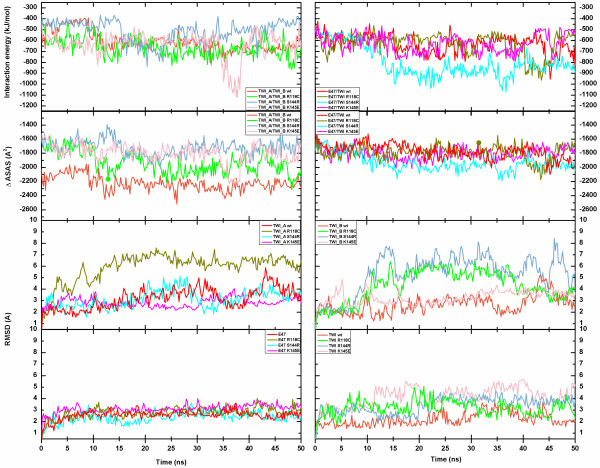
**Structural stability assessment during the MD simulations. **The first row presents the interaction potential energy assessed for all dimers using Coulomb and Lennard-Jones terms. The images in the left column depict the wt and mutant homodimers (TWI_A/TWI_B), and the images in the right column depict the wt and mutant heterodimers (E47/TWI). The second row represents the variation of the solvent accessible surface area (SASA) for the dimers and was calculated by subtracting the sum of the SASA of the individual monomers from the SASA of their respective dimer [ΔSASA = TWI_A/TWI_B SASA – (TWI_A SASA + TWI_B SASA)]. The ΔSASA for the homodimers is presented in the left column and the heterodimer value is presented in the right column. The RMSD analysis (third and fourth row) for all of the monomers was calculated as a function of the backbone structure and separately according to the monomers. The third row images represent homodimer monomers (in the left column TWI_A and in the right column TWI_B wt and mutant monomers), and on the fourth row the heterodimer monomers are depicted (E47 monomers in the left column and TWI wt and mutant monomers in the right column). Å – angstrom (10^-10^ m); kJ – kilojoule (10^3^joules); mol - 6,02 · 10^23^ particles; ns – nanoseconds (10^-9^ s).

Due to protein stability, out of the 50 ns of simulation time, only the last 30 ns were subjected to complete analysis. The interaction potential energy between monomers remained constant along the simulation time for wt structures. Out of all mutated dimers, the E47/TWI S144R and TWI_A/TWI_B K145E dimers presented the lowest interaction energy level (Figure 4).

All of the time evolution analysis was performed using the GROMACS package taking into account all of the atoms, the backbone and the Cα atoms of the structures to ascertain whether there was a significant movement of the residues. Once the difference between the structures with and without side-chains was within the expected range, we decided to investigate the backbone.

The root mean square deviation (RMSD – Figure 4) and the radii of gyration (Rg) analysis of the protein, taking the equilibrated configuration (after minimization and before MD simulation) as reference, indicated that the wt dimers presented similar deviations over time. The Rg analysis did not present variations that were higher than 2 Å, which indicated that there were no major deformations on the protein as a whole or on any of the domains (Additional file 4: Figure S1). Interestingly, the RMSD analysis indicated an average deviation of up to 6 Å for homodimers and 3 Å for heterodimers, and the mutated homodimers presented values that were higher than the wt, demanding further meticulous investigations. The solvent accessible surface area variation (ΔSASA) was calculated by subtracting the sum of the SAS of the individual monomers from the SAS of their respective dimer. Negative ΔSASA values indicate that the association of both monomers resulted in a good mesh. The evolution of ΔSASA indicated unsigned variations greater than 1700 Å^2^ (Table 2) and presented a constant area during the simulation time (Figure 4). All of the heterodimers presented a similar ΔSASA. The wt homodimer presented a lower value when compared with mutated models and exhibited a more compact conformation between monomers. The ΔSASA time evolution was compatible with the energy variation, where the lowest energy corresponded to the most compacted form of the analyzed dimer (Figure 4), along with the formation of additional hydrogen bonds (data not shown).

**Table 2:** Average values of geometrical property statistics during the last 30 ns of MD simulations

	**Model**	**ΔSASA (A**^**2**^**)**	**RMSD (A)**										**Rg (A)**	
			**Total**		**Basic**		**Helix I**		**Loop**		**Helix II**		**Basic**	
			**mon1**	**mon2**	**mon1**	**mon2**	**mon1**	**mon2**	**mon1**	**mon2**	**mon1**	**mon2**	**mon1**	**mon2**
HOMODIMER (TWI_A/TWI_B)	Wt	-2266.56 (70.3)	3.64 (0.72)	2.99 (0.77)	2.32 (0.78)	3.01 (0.53)	0.43 (0.09)	0.39 (0.08)	2.02 (0.26)	1.21 (0.14)	0.97 (0.42)	1.01 (0.15)	8.35 (0.30)	7.46 (0.47)
	R118C	-2007.47 (111)	6.47 (0.45)	4.85 (1.02)	2.96 (0.19)	3.75 (0.38)	0.48 (0.11)	1.22 (0.36)	1.37 (0.12)	2.78 (0.32)	1.83 (0.56)	1.90 (0.47)	7.53 (2.51)	7.93 (0.64)
	S144R	-1743.64 (98.6)	3.50 (0.66)	6.09 (0.95)	2.51 (0.78)	3.06 (0.77)	0.52 (0.16)	0.64 (0.10)	1.37 (0.19)	1.54 (0.22)	0.90 (0.22)	0.97 (0.23)	7.83 (0.51)	7.60 (0.51)
	K145E	-1846.84 (134)	2.86 (0.40)	3.43 (0.41)	3.40 (0.78)	3.27 (0.38)	1.59 (0.13)	1.39 (0.13)	2.18 (0.16)	3.94 (3.94)	2.11 (0.42)	2.26 (0.26)	7.28 (0.25)	7.22 (0.28)
HETERODIMER (E47/TWI)	Wt	-1829.10 (88.8)	2.64 (0.25)	2.45 (0.57)	3.49 (0.24)	2.29 (0.53)	0.72 (0.13)	0.39 (0.08)	1.42 (0.25)	1.66 (0.24)	1.08 (2.29)	0.90 (0.26)	7.75 (0.68)	7.70 (0.44)
	R118C	-1797.56 (111.7)	2.91 (0.34)	3.27 (0.62)	3.80 (0.24)	2.80 (0.38)	1.27 (0.30)	1.22 (0.36)	1.60 (0.34)	1.43 (2.28)	0.60 (0.11)	0.96 (0.24)	7.20 (0.22)	7.43 (0.50)
	S144R	-1977.27 (78.2)	2.46 (0.37)	3.85 (0.35)	2.44 (0.22)	2.29 (0.55)	0.73 (0.11)	0.64 (0.10)	0.76 (0.13)	1.63 (0.15)	1.08 (0.14)	1.48 (0.15)	7.89 (0.39)	7.33 (0.27)
	K145E	-1902.91 (73)	3.23 (0.25)	4.46 (0.56)	4.02 (0.25)	5.39 (0.46)	2.22 (0.18)	1.39 (0.13)	2.21 (0.18)	2.68 (0.27)	2.21 (0.21)	2.11 (0.29)	8.19 (0.26)	8.07 (0.44)

Standard deviations are shown in parentheses. Å – angstrom (10^-10^ m); wt – wild-type; Rg – radius of gyration; RMSD – root-mean-square-deviation, taking as reference their respective starting structures. mon1 is monomer TWI_A in the homodimer and monomer E47 in the heterodimer; mon2 is monomer TWI_B in the homodimer and monomer TWI in the heterodimer.

The analysis of the secondary structure of the protein bHLH dimer throughout the analysis using the DSSP program did not present significant variations (Additional file 5: Figure S2). Therefore, all of the dimers did not exhibit large variations with respect to exposed residues and kept their structures folded.

The initial profile and behavior during the simulation of the wt and the mutated residues regarding hydrophobic/hydrophilic SASA, volume and average area were assessed (Additional file 3: Table S3). The ratio between the mean and the equilibrated structure was calculated (indicated in parentheses), and the values less than 1 (in bold) indicated that the parameter decreased when compared with the reference. The hydrophilic SASA of C118 of the R118C mutant form decreased for the homo- and heterodimers and increased for R144 of the S144R mutant, indicating that C118 became less hydrophilic (and charged) and R144 turned into a more hydrophilic residue. The K145E mutation also changed the residue to a more hydrophilic one.

The decrease of the total SASA and average area on the C118 residue of 90 Å^2^ and the increase of 100 Å^2^ on the R144 residue were conserved throughout the simulation for both the homodimers and heterodimers. For the E145 mutation, the area remained constant and did not differ between the wt and the mutated residue for both dimers (Additional file 3: Table S3).

**Figure 5:**
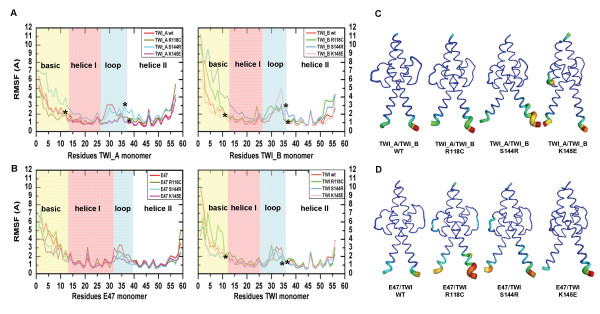
**Comparison of the atomic fluctuations per residue for the last 30 ns of the simulation.** The RMSF values for each monomer of the homodimer and heterodimer are displayed in (**A**) and (**B**), respectively. Asterisks (*) indicate the mutated residues of the TWIST1 monomer. The b-factors represented in cartoon form for the homo- and heterodimers (the wt and mutants) are displayed in (**C**) and (**D**), respectively. The most fluctuating residues are colored in red (terminal residues), and the remainder of the dimer is in light blue (the most stable), in accordance with the observed fluctuations observed. Å – angstrom (10^-10^ m);ns – nanoseconds (10^-9^ s).

The root-mean-square-fluctuation (RMSF) for each residue was calculated and plotted in Figure 5. The residue numbers were labeled according to their alignment (Figure 2). Fluctuations of up to 10 Å were found at both ends of all of the monomers and in the loop region, which were completely exposed to the solvent, thus presenting higher mobility. The basic domain of the homo- and heterodimers displayed the highest fluctuation, and the mutated proteins exhibited more variations than the wt (Figure 5A). The higher fluctuation values at the ends (the basic and C-terminus) were likely observed because of the absence of the remainder of the protein, which is necessary to stabilize the 3D structure. Calculated b-factor parameters (Figure 5B) also exposed this fact, and the high fluctuation of the TWIST1 monomer basic domain of the R118C heterodimer is represented with a thicker, red tube.

### The basic domain presents distinct behavior compared with other domains

**Figure 6:**
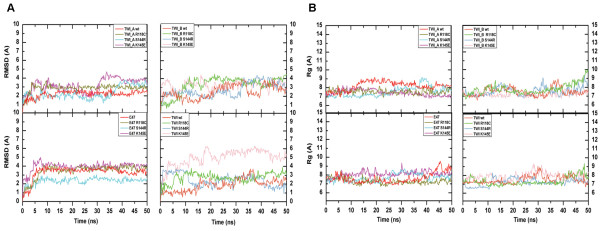
**RMSD and Rg of the basic region of each monomer for 50 ns of simulation time. **(**A**) The RMSD and (**B**) the Rg behavior of all dimers. The minimized structures (t = 0 ns) were taken as a reference. The upper images represent the homodimers (TWI_A and TWI_B) and the bottom images correspond to the heterodimers (E47 and TWI). Å – angstrom (10^-10^ m);ns – nanoseconds (10^-9^ s).

To perform a better analysis, the monomers were divided into four regions—basic, helix I, loop and helix II—and the RMSD was plotted as a function of time for each region. The backbone RMSD of the basic domain for both of the dimers indicated a fluctuation of up to 4 Å, in contrast to the first helix (up to 2 Å), the loop (up to 3 Å) and the second helix (up to 3 Å) (data not shown), confirming that this region presented the highest deviation from the reference equilibrated structure. Despite having taken the reference structure, all of the systems that were simulated required a substantial amount of time to become organized and to become structurally stable (approximately 20 ns). On the other hand, the Rg for all of the atoms for the same region did not present a considerable redistribution of the atomic positions (Figure 6 and Table 2).

The minimum distance between the centers of mass for the regions presented higher values for the basic domain (data not shown), confirming the previous analysis. The mutated proteins presented RMSD values that were higher than for the wt, except for the S144R homodimer, which presented values that were similar to the wt homodimer.

### Tracking the cavity gap motion by essential dynamic analysis

**Figure 7:**
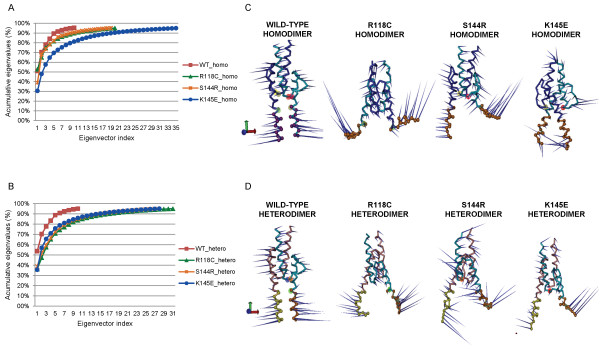
**Collective motion analysis. **(**A**) and (**B**) represent the percentage of cumulative eigenvalues as a function of eigenvector indices for the homodimers and heterodimers, respectively. (**C**) and (**D**) represent the porcupine plot of the first eigenvector of the homodimer and heterodimer, respectively. The basic domain is colored in orange for the TWIST1 monomers and in yellow for the E47 monomer. The cones point in the direction of indication mode of atomic movement, and the amplitude of the motion is represented by the length of the cone.

To identify the overall patterns of the motions and to visualize the high mobility of the basic domain of the TWIST1 dimers, we used principal component analysis (PCA), which relies on the hypothesis that major collective modes of fluctuation dominate the functional dynamics of a biomolecular system. PCA was performed on the trajectory data using the mass-weighted covariance matrix of the atomic coordinates, where the eigenvectors give the direction of the motion and the eigenvalues account for the associated extent of the motions (a RMSF of the collective motion) [38]. The results of the PCA are presented in Figure 7, where the percentages of cumulative eigenvalues are plotted in a function of eigenvector index (7A and 7B) and where the movements projected along the first eigenvector for the wt and each mutant are represented with porcupine plots (7 C and 7D). The cones point in the direction that the atoms move while the length of the cone represents the amplitude (7 C and 7D). The images (7A and 7B) display the computation of the relative contribution to protein fluctuation for each eigenvector (also shown in Additional file 6: Table S4), and the first three eigenvectors were responsible for more than 50% of the collective motion for all dimers. The first eigenvector of the wt dimers was charged with approximately 50% of motion, while for the mutant dimers, it was accountable for 40% at most. The R118C homodimer was an exception because the first eigenvector corresponded with up to 53% of motion (Additional file 6: Table S4). This most likely occurred because the mutated residue promoted instability of the basic domain, which led to the flexibility of the protein (Figure 7C). The four most representative collective motions (Additional file 7: Figure S3) for the mutated dimers reinforce the observation that the basic domain demonstrated an aberrant motion, opening the cleft in different directions and with different amplitudes (which was not observed in wt forms). The fluctuations of the residues belonging to the basic domain of TWIST1 monomers were highlighted by RMSD and RMSF analyses. Yet, the orientation of the collective motion of the basic domain and its amplitude were better evaluated by the study of the porcupine plots.

## Discussion

Currently, no 3D structure of TWIST1 is available. Therefore, the aim of this study was to predict this structure along with important mutations in 3 regions by using the homology modeling technique and to study the behavior of the structures in aqueous solution. No entire 3D structure of a eukaryotic transcription factor is present in the Protein Data Bank, which is most likely because most transcription factors with modular structures commonly possess one or more intrinsically disordered (ID) regions/domains, usually in terminal tails and linker regions between domains [39]. For human TWIST1, there is a large disordered region in the N-terminus (residues 3 to 102) that is known to interact with p300 and HAT, among other proteins [40]. However, this interaction has not been demonstrated *in vivo* yet. The disordered region contains 2 nuclear localization signals [41]. The C-terminal region of TWIST1, which is highly conserved among vertebrates and contains a “twist-box” (WR motif) [42], also presented a large ID region that is intercalated with α-helix domains.

The bHLH domain of the TWIST1 protein is of special interest because some of the most frequent mutations described for SCS occur in this domain. In addition, the domain is closely related to transcription factor function. The high sequence similarity of the bHLH domain among the various proteins of the same family and a large amount of experimental structural data allowed us to model the bHLH domain of TWIST1 and the R118C, S144R and K145E mutations in TWIST1 monomers by comparative modeling. There is a high level of conservation for human R118 and K145 across species, and the modification to a non-conserved residue could explain the loss of DNA-binding capacity, which is critical to TF function. The S144 is less conserved among species, although it is present in more than 30% of the identified bHLH TF family members [43]. The choice of E47 as a dimerization partner was based on evidence that this partnership occurs in patients suffering the genetic disorder SCS [44]. Recent works have shown that TWIST1 transcription complexes co-precipitate with E12 and E47 [45]. In addition, other bHLH proteins may be TWIST1 partners, depending on the tissue and environment conditions, and will affect the expression of different targets.

All of the mutations that were described and assessed in this study modify the charge and volume of the residue side chains. The dimers also presented an accessible surface that is smaller for the wild-type proteins, demonstrating that, in mutant proteins, a higher area is exposed. The heterodimers presented better behavior in all analyzed simulation conditions. This performance is accordance with the literature, which describes heterodimers formed by E47 and other bHLH monomer as more stable than their respective homodimers [46,47]. The stability observed in the presence of the E47 monomer may be because helix 1 of E47 is one turn longer than TWIST1 (and MyoD), which results in an increase in the buried surface at the dimer interface. In addition, the loop region may form a network of hydrogen bonds that bridges helices 1 and 2, stabilizing its fold, which most likely contributes to the stability of the E47 dimer. The E47 protein is stably folded and dimeric in the absence of DNA binding, whereas MyoD, despite of its sequence similarity to E47, presents a more unstable dimer [47]. The basic domain motion for all dimers detected in our study supports the idea that transcription factors should present an adaptable DNA-binding region in a way that fits different target genes. The wider range of motion observed mainly for the R118C homodimer may be due to instability caused by this mutated residue. The other mutated dimers also presented a wider motion than the wt dimer but on a smaller scale than TWI_A/TWI_B R118C. Our results support El Ghouzzi´s suggestion of why these mutations impair TWIST1 binding to DNA [48]. The author used an electrophoretic mobility shift assay to demonstrate the loss of binding capacity for wt and mutated dimers. The previous conclusion was made based upon the crystallographic structure of the bHLH family member MYOD, as the basic region of both MYOD and TWIST1 present high sequence identity. The modifications described by El Ghouzzi were applied to MYOD by rotating the side-chain of the mutated residue to infer the consequences to DNA-binding. It is noteworthy that this modification was performed on a static structure, without energy minimization and molecular dynamics simulation, which we have accomplished here.

## Conclusions

Both the TWIST1 homodimer and the E47/TWIST1 heterodimer bHLH models presented no major deformations in their structures or high amplitude movements except for the basic region. The basic region movements were accentuated in the homodimers. This behavior could be explained by the fact that this region is where the protein binds to the DNA molecule; therefore, a high degree of flexibility is adequate and suitable for fitting. The dimers harboring the mutations R118C, S144R and K145E presented RMSD values that were higher than the corresponding ones for the wild-type dimers, thus verifying the observed flexibility of this domain. It was also observed that the aberrant movement may be the reason why these dimers fail to bind to target DNA in a stable way. This hypothesis will be addressed by simulating these mutated dimers in complex with target DNA for a longer period.

## Competing interests

The authors declare that they have no competing interests.

## Authors’ contributions

Conceived and designed the experiments: AMM, ERC and EA. Performed the experiments: AMM, JHMS, and ERC. Analyzed the data: AMM, JHMS, ALM, ERC and EA. Wrote the paper: AMM, JHMS, ALM, ERC and EA. All authors read and approved the final manuscript.

## Supplementary Material

Additional file 1 **Table S1. **Final configuration for molecular dynamics simulation. Cl- – chloride ions. A – angstrom; wt – wild-type. Click here for file

Additional file 2 **Table S2.** Available templates with similar structure with TWIST1 sequence. The E47/NeuroD1 complex (accession number 2QL2) used as a template for comparative modeling corresponded to chains C and D, respectively. The capital letters in parentheses correspond to the chain in the crystal. *Mm – Mus musculus*; *Hs – Homo sapiens*; NMR – nuclear magnetic resonance; Å – angstrom (10^-10^ m). Click here for file

Additional file 3 **Table S3. **Area variation between the wild-type and mutated residues. The TWI_A and TWI_B columns represent homodimer monomers 1 and 2, while the TWI columns correspond to the TWIST1 monomer of the heterodimer. The ratio between the mean and the equilibrated structures is in parentheses. The bolded values decreased throughout the simulation; Å – angstrom (10^-10^ m). Click here for file

Additional file 4 **Figure S1. **Radius of gyration for each domain of TWIST1 homo- and heterodimers wt and mutants. Rg analysis was performed for each domain: (A) basic, (B) helix I, (C) loop and (D) helix II. The upper images correspond to the homodimers and the lower images correspond to the heterodimers. Å – angstrom (10^-10^ m);ns – nanoseconds (10^-9^ s). Click here for file

Additional file 5 **Figure S2. **Secondary structure analysis (DSSP) for each dimer in function of time simulation. All eight dimers were assessed for secondary structure over simulation, and the color coding indicates the conformation of the residue sequence. ns – nanoseconds (10^-9^ s). Click here for file

Additional file 6 **Figure S3. **Porcupine plots of the four most representative collective motions of all analyzed dimers. The porcupine plots of the four most representative collective motions and the percentage of the motion for each dimer are shown. The homodimers are in blue boxes (TWI_A/TWI_B wt, R118C, S144R and K145E) and the heterodimers are in red boxes (E47/TWI wt, R118C, S144R and K145E). The E47 monomer is represented in pink. The cones point in the direction of atomic movement along the indicated mode of motion, and the amplitude of the motion is represented by the length of the cone. Click here for file

Additional file 7 **Table S4. **The contribution of the first 10 modes to the total motion of TWIST1 dimers. The percentage of motion is given by the absolute percentage (%) and the cumulative normalized eigenvalues (CNF) are the sum of the eigenvector percentages. The first three eigenvectors were responsible for more than 50% of the motion for all dimers. eigen – eigenvalue; wt – wild-type; CNF – percentage of cumulative normalized eigenvalues. Click here for file
