# Supplementary material for: Computational modeling of the bHLH domain of the transcription factor TWIST1 and R118C, S144R and K145E mutants
Source: BMC Bioinformatics. 2012 Jul 28;13:184. doi: 10.1186/1471-2105-13-184 (PMC3507644; doi:10.1186/1471-2105-13-184)
Supplement: Additional file 1 — Table S1. Final configuration for molecular dynamics simulation. Cl- – chloride ions. A – angstrom; wt – wild-type. [file 1471-2105-13-184-S1.doc]

**Table S1: Final configuration for molecular dynamics simulation**.

| Molecules | TWI_A/TWI_B WT | TWI_A/TWI_B R118C | TWI_A/TWI_B S144R | TWI_A/TWI_B K145E | E47/TWI  WT | E47/TWI R118C | E47/TWI S144R | E47/TWI K145E |
| --- | --- | --- | --- | --- | --- | --- | --- | --- |
| Number of atoms (-H_2_O,Cl^-^) | 1925 | 1899 | 1951 | 1911 | 1962 | 1949 | 1975 | 1955 |
| Number of H_2_O molecules | 8841 | 8843 | 8824 | 8839 | 8828 | 8828 | 8827 | 8832 |
| Box dimensions (Å^3^) | 60x60x80 | 60x60x80 | 60x60x80 | 60x60x80 | 60x60x80 | 60x60x80 | 60x60x80 | 60x60x80 |
| Residual charge neutralization (Cl^-^) | 12 | 10 | 14 | 8 | 11 | 9 | 12 | 9 |
| Simulation time | 50 ns | 50 ns | 50 ns | 50 ns | 50 ns | 50 ns | 50 ns | 50 ns |

Cl- chloride ions. Å-angstrom; wt-wild-type.
