# Supplementary material for: Computational modeling of the bHLH domain of the transcription factor TWIST1 and R118C, S144R and K145E mutants
Source: BMC Bioinformatics. 2012 Jul 28;13:184. doi: 10.1186/1471-2105-13-184 (PMC3507644; doi:10.1186/1471-2105-13-184)
Supplement: Additional file 2 — Table S2. Available templates with similar structure with TWIST1 sequence. The E47/NeuroD1 complex (accession number 2QL2) used as a template for comparative modeling corresponded to chains C and D, respectively. The capital letters in parentheses correspond to the chain in the crystal. Mm – Mus musculus; Hs – Homo sapiens; NMR – nuclear magnetic resonance; Å – angstrom (10-10 m). [file 1471-2105-13-184-S2.doc]

**Table S2: Available templates with similar structure with TWIST1 sequence.**

| PDB | ID | Species | E-Value | Identity | Length | Bits(score) | Resolution | Method |
| --- | --- | --- | --- | --- | --- | --- | --- | --- |
| 2QL2 (A,B,C,D) | E47/NeuroD1 + DNA | *Mm* | 1e-08 (B,D)/  1.4 (A,C) | 47% - 28/59 (B,D)/  44% - 15/34 (A,C) | 60 | 54.3 (B,D)/  27.3 (A,C) | 2.5 A | X-ray crystallography |
| 1MDY (A,B,C,D) | MyoD/MyoD + DNA | *Mm* | 5e-05 | 37% - 21/56 | 62 (B,C,D)/  68 (A) | 42.0 | 2.8 A | X-ray crystallography |
| 1R05  (A,B) | Max/Max | *Hs* | 3e-04 | 31% - 17/54 | 87 | 39,7 | ---- | NMR |
| 1NKP (A,B,D,E) | Myc/Max + DNA | *Hs* | 3e-04 (B,E)/  0.013 (A,D) | 33% - 7/51(B,E)/  42% - 2/52 (A,D) | 83 (B,E)/  88 (A,D) | 39.3 (B,E)/  34.3 (A,D) | 1.8 A | X-ray crystallography |
| 1HLO  (A,B) | Max/Max + DNA | *Hs* | 3e-04 | 33% - 17/51 | 80 | 39.3 | 2.8 A | X-ray crystallography |
| 1NLW  (B,E) | Mad/Max + DNA | *Hs* | 3e-04 | 33% - 17/51 | 76 | 39.3 | 2.0 A | X-ray crystallography |

Source: BLASTp/PDB results and Brookhaven database.
