# Supplementary material for: Computational modeling of the bHLH domain of the transcription factor TWIST1 and R118C, S144R and K145E mutants
Source: BMC Bioinformatics. 2012 Jul 28;13:184. doi: 10.1186/1471-2105-13-184 (PMC3507644; doi:10.1186/1471-2105-13-184)
Supplement: Additional file 3 — Table S3. Area variation between the wild-type and mutated residues. The TWI_A and TWI_B columns represent homodimer monomers 1 and 2, while the TWI columns correspond to the TWIST1 monomer of the heterodimer. The ratio between the mean and the equilibrated structures is in parentheses. The bolded values decreased throughout the simulation; Å – angstrom (10-10 m). [file 1471-2105-13-184-S3.doc]

**Table S3: Area variation between the wild-type and mutated residues.**

| Area | R118 | | | C118 | | | S144 | | | R144 | | | K145 | | | E145 | | |
| --- | --- | --- | --- | --- | --- | --- | --- | --- | --- | --- | --- | --- | --- | --- | --- | --- | --- | --- |
|  | TWI_A | TWI_B | TWI | TWI_A | TWI_B | TWI | TWI_A | TW_IB | TWI | TWI_A | TWI_B | TWI | TWI_A | TWI_B | TWI | TWI_A | TWI_B | TWI |
| hydrophobic SASA (Å^2^) | 89.1 | 89.1 | 82.5 | 43.4 | 43.4 | 46.7 | 49.5 | 52.8 | 59.4 | 95.7 | 92.4 | 82.5 | 125.5 | 102.4 | 118.8 | 39.6 | 36.3 | 36.3 |
|  | (1.01) | (1.01) | (1.09) | (1.12) | (1.16) | (1.07) | (1.10) | (1.09) | **(0.98)** | **(0.96)** | **(0.98)** | (1.11) | (1.01) | (1.23) | (1.06) | (1.09) | (1.02) | (1.03) |
| hydrophilic SASA (Å^2^) | 202.5 | 206.3 | 183.9 | 166.3 | 164.2 | 159.4 | 134.6 | 134.3 | 124.8 | 201.8 | 196.4 | 207.8 | 145.2 | 141.7 | 148.5 | 209.0 | 204.9 | 216.4 |
|  | (1.01) | **(0.98)** | (1.10) | **(0.96)** | **(0.96)** | **(0.99)** | **(0.99)** | **(0.96)** | (1.05) | (1.01) | (1.06) | **(0.97)** | **(0.98)** | **(0.91)** | (1.01) | **(0.91)** | **(0.98)** | **(0.92)** |
| total SASA (Å^2^) | 291.7 | 295.4 | 266.5 | 209.7 | 207.7 | 206.2 | 184.2 | 187.1 | 184.2 | 297.6 | 288.8 | 290.4 | 270.7 | 244.1 | 267.4 | 248.6 | 241.2 | 252.7 |
|  | (1.01) | **(0.98)** | (1.10) | **(0.99)** | **(0.76)** | (1.01) | (1.02) | **(0.99)** | (1.01) | **(0.99)** | (1.02) | (1.01) | **(0.99)** | (1.10) | (1.00) | **(0.94)** | **(0.98)** | **(0.94)** |
| Average area (Å^2^) | 291.8 | 294.4 | 285.0 | 207.3 | 207.7 | 207.2 | 183.9 | 186.8 | 189.2 | 291.8 | 296.2 | 288.8 | 275.7 | 252.8 | 266.1 | 238.5 | 246.9 | 237.8 |
|  | (1.00) | **(0.99)** | (1.02) | (1.00) | (1.00) | (1.00) | (1.01) | **(0.99)** | **(0.98)** | (1.01) | **(0.99)** | (1.01) | **(0.97)** | (1.06) | (1.00) | **(0.98)** | **(0.96)** | **(0.99)** |
