# Supplementary figures and images for: Computational modeling of the bHLH domain of the transcription factor TWIST1 and R118C, S144R and K145E mutants
Source: BMC Bioinformatics. 2012 Jul 28;13:184. doi: 10.1186/1471-2105-13-184 (PMC3507644; doi:10.1186/1471-2105-13-184)

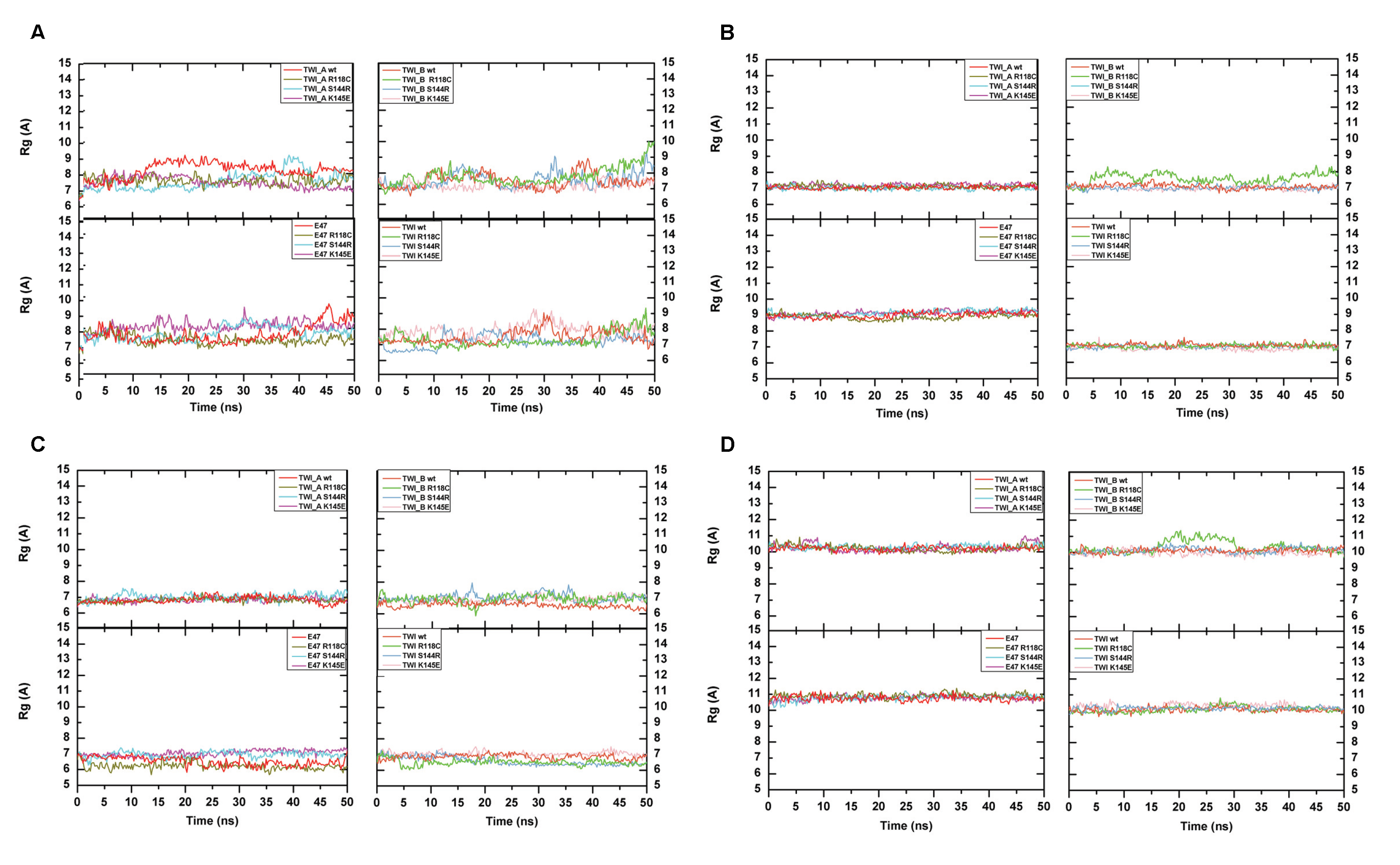

Supplement: Additional file 4 — Figure S1. Radius of gyration for each domain of TWIST1 homo- and heterodimers wt and mutants. Rg analysis was performed for each domain: (A) basic, (B) helix I, (C) loop and (D) helix II. The upper images correspond to the homodimers and the lower images correspond to the heterodimers. Å – angstrom (10-10 m);ns – nanoseconds (10-9 s). [file 1471-2105-13-184-S4.tiff]

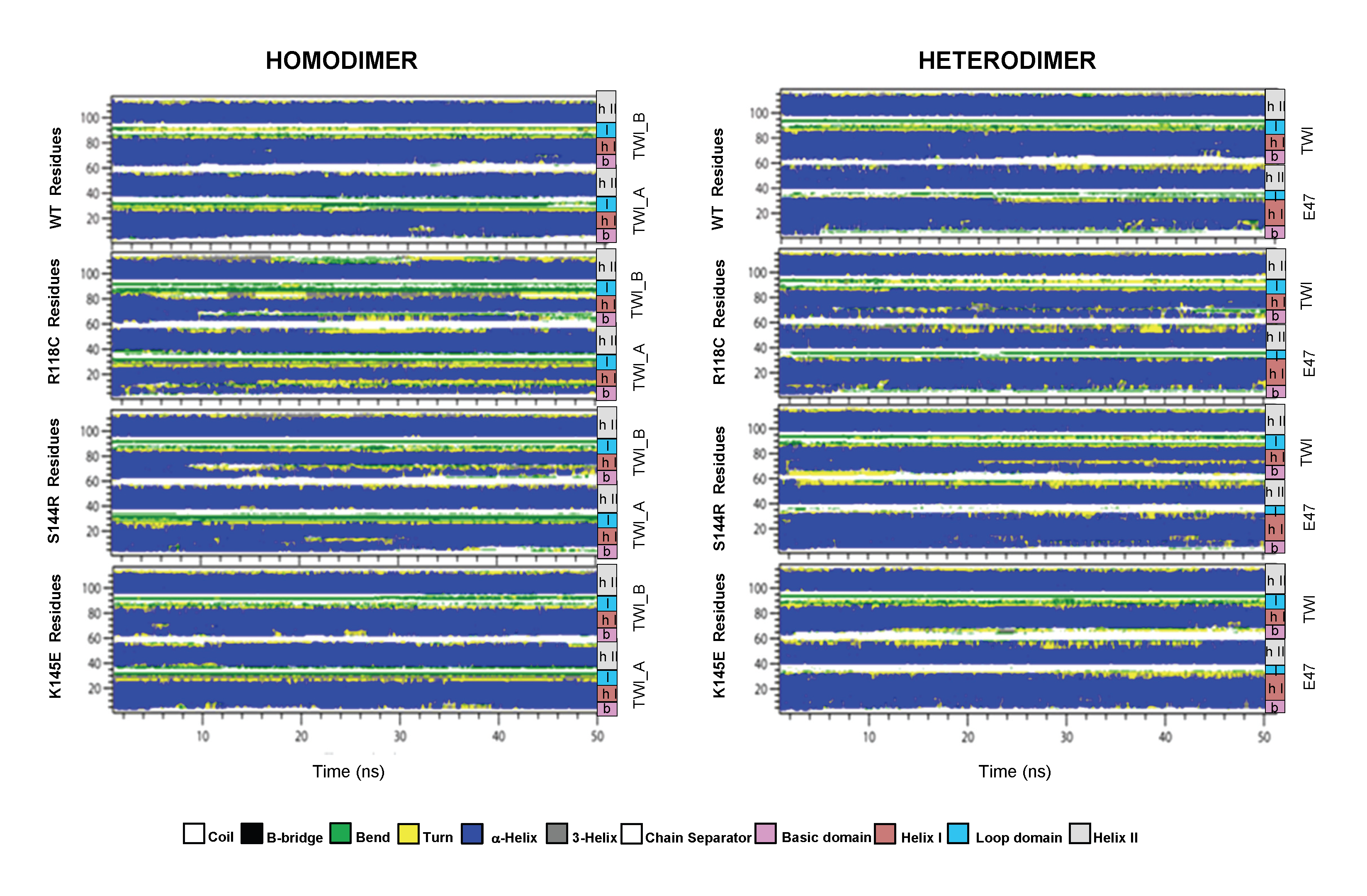

Supplement: Additional file 5 — Figure S2. Secondary structure analysis (DSSP) for each dimer in function of time simulation. All eight dimers were assessed for secondary structure over simulation, and the color coding indicates the conformation of the residue sequence. ns – nanoseconds (10-9 s). [file 1471-2105-13-184-S5.tiff]

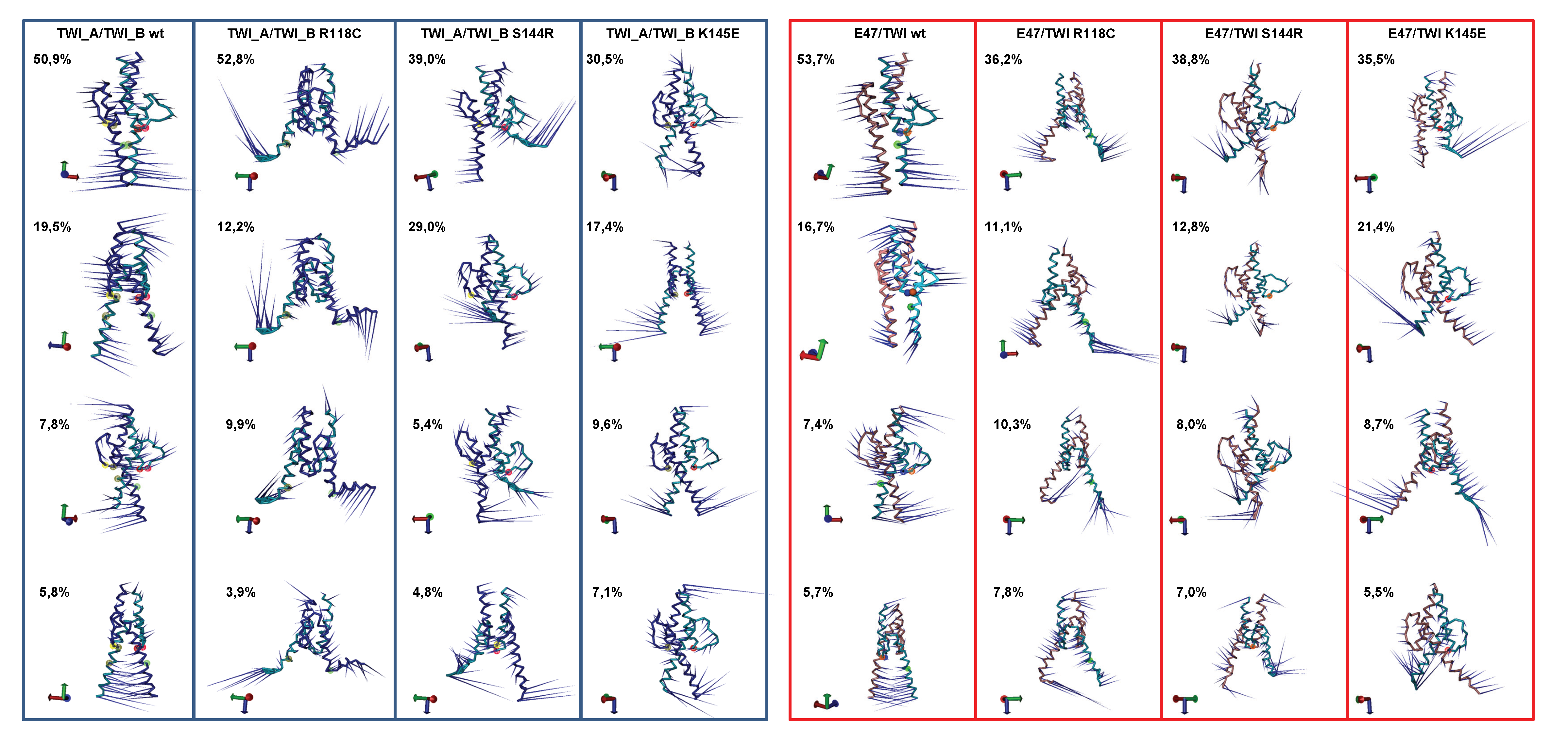

Supplement: Additional file 7 — Table S4. The contribution of the first 10 modes to the total motion of TWIST1 dimers. The percentage of motion is given by the absolute percentage (%) and the cumulative normalized eigenvalues (CNF) are the sum of the eigenvector percentages. The first three eigenvectors were responsible for more than 50% of the motion for all dimers. eigen – eigenvalue; wt – wild-type; CNF – percentage of cumulative normalized eigenvalues. [file 1471-2105-13-184-S7.tiff]
