# Supplementary material for: Computational modeling of the bHLH domain of the transcription factor TWIST1 and R118C, S144R and K145E mutants
Source: BMC Bioinformatics. 2012 Jul 28;13:184. doi: 10.1186/1471-2105-13-184 (PMC3507644; doi:10.1186/1471-2105-13-184)
Supplement: Additional file 6 — Figure S3. Porcupine plots of the four most representative collective motions of all analyzed dimers. The porcupine plots of the four most representative collective motions and the percentage of the motion for each dimer are shown. The homodimers are in blue boxes (TWI_A/TWI_B wt, R118C, S144R and K145E) and the heterodimers are in red boxes (E47/TWI wt, R118C, S144R and K145E). The E47 monomer is represented in pink. The cones point in the direction of atomic movement along the indicated mode of motion, and the amplitude of the motion is represented by the length of the cone. [file 1471-2105-13-184-S6.doc]

**Table S4: The contribution of the first 10 modes to the total motion of TWIST1 dimers.**

| INDEX | HOMODIMER | | | | | | | | | | | | HETERODIMER | | | | | | | | | | | |
| --- | --- | --- | --- | --- | --- | --- | --- | --- | --- | --- | --- | --- | --- | --- | --- | --- | --- | --- | --- | --- | --- | --- | --- | --- |
|  | WT | | | R118C | | | S144R | | | K145E | | | WT | | | R118C | | | S144R | | | K145E | | |
|  | eigenv | % | CNF | eigenv | % | CNF | eigenv | % | CNF | eigenv | % | CNF | eigenv | % | CNF | eigenv | % | CNF | eigenv | % | CNF | eigenv | % | CNF |
| 1 | 3.901 | 50.9% | 50.9% | 6.501 | 52.8% | 52.76% | 4.93 | 39.0% | 39.0% | 1.221 | 30.5% | 30.5% | 4.2404 | 53.7% | 53.7% | 2.361 | 36.2% | 36.2% | 1.904 | 38.8% | 38.8% | 1.944 | 35.5% | 35.5% |
| 2 | 1.491 | 19.5% | 70.4% | 1.507 | 12.2% | 65.0% | 3.668 | 29.0% | 68.0% | 0.698 | 17.4% | 48.0% | 1.3228 | 16.7% | 70.4% | 0.725 | 11.1% | 47.3% | 0.626 | 12.8% | 51.6% | 1.169 | 21.4% | 56.9% |
| 3 | 0.595 | 7.8% | 78.2% | 1.22 | 9.9% | 74.9% | 0.684 | 5.4% | 73.4% | 0.386 | 9.6% | 57.6% | 0.5829 | 7.4% | 77.8% | 0.673 | 10.3% | 57.6% | 0.394 | 8.0% | 59.7% | 0.475 | 8.7% | 65.6% |
| 4 | 0.448 | 5.8% | 84.0% | 0.478 | 3.9% | 78.8% | 0.607 | 4.8% | 78.2% | 0.285 | 7.1% | 64.7% | 0.4496 | 5.7% | 83.5% | 0.509 | 7.8% | 65.4% | 0.344 | 7.0% | 66.7% | 0.299 | 5.5% | 71.1% |
| 5 | 0.413 | 5.4% | 89.4% | 0.357 | 2.9% | 81.7% | 0.5 | 4.0% | 82.1% | 0.193 | 4.8% | 69.5% | 0.4181 | 5.3% | 88.8% | 0.359 | 5.5% | 70.9% | 0.3 | 6.1% | 72.8% | 0.265 | 4.9% | 75.9% |
| 6 | 0.163 | 2.1% | 91.5% | 0.336 | 2.7% | 84.4% | 0.461 | 3.6% | 85.8% | 0.127 | 3.2% | 72.7% | 0.1683 | 2.1% | 90.9% | 0.23 | 3.5% | 74.4% | 0.18 | 3.7% | 76.5% | 0.164 | 3.0% | 78.9% |
| 7 | 0.098 | 1.3% | 92.8% | 0.217 | 1.8% | 86.2% | 0.183 | 1.4% | 87.2% | 0.103 | 2.6% | 75.3% | 0.1243 | 1.6% | 92.5% | 0.18 | 2.8% | 77.1% | 0.135 | 2.7% | 79.2% | 0.124 | 2.3% | 81.2% |
| 8 | 0.092 | 1.2% | 94.0% | 0.164 | 1.3% | 87.5% | 0.176 | 1.4% | 88.6% | 0.098 | 2.5% | 77.7% | 0.094 | 1.2% | 93.7% | 0.166 | 2.5% | 79.7% | 0.118 | 2.4% | 81.6% | 0.103 | 1.9% | 83.0% |
| 9 | 0.055 | 0.7% | 94.7% | 0.152 | 1.2% | 88.7% | 0.144 | 1.1% | 89.8% | 0.076 | 1.9% | 79.6% | 0.0622 | 0.8% | 94.5% | 0.155 | 2.4% | 82.1% | 0.097 | 2.0% | 83.6% | 0.092 | 1.7% | 84.7% |
| 10 | 0.048 | 0.6% | 95.3% | 0.133 | 1.1% | 89.8% | 0.115 | 0.9% | 90.7% | 0.066 | 1.7% | 81.3% | 0.0547 | 0.7% | 95.2% | 0.11 | 1.7% | 83.7% | 0.07 | 1.4% | 85.0% | 0.073 | 1.3% | 86.0% |
| total |  | 95.3% |  |  | 89.8% |  |  | 90.6% |  |  | 81.3% |  |  | 95.2% |  |  | 83.8% |  |  | 84.9% |  |  | 86.2% |  |
